# Mentorship in a Surgical Residency: A Comprehensive Review of the Literature

**DOI:** 10.7759/cureus.43422

**Published:** 2023-08-13

**Authors:** Kevin Steelman, Dominik Fleifel, Muhammad Waheed, Rahul Vaidya

**Affiliations:** 1 Orthopaedic Surgery, Wayne State University Detroit Medical Center, Detroit, USA

**Keywords:** graduate medical education, stages of mentorship, residency, surgery, mentorship

## Abstract

Mentorship in surgical training is an experience that extends beyond the teacher-student interaction. Effective mentorship is crucial in surgical training and requires ongoing support at all stages of graduate surgical education, particularly in the context of busy surgical residency programs. It is important to recognize that mentors and mentees may have different styles of learning and teaching, making it essential to discuss and review these approaches to ensure effective mentorship. By acknowledging these differences and developing a supportive mentorship program that addresses them, surgical residents can receive the guidance they need to progress successfully through their training and prepare for independent practice. This review provides a comprehensive analysis of mentorship styles in various surgical training residencies. By including 30 publications, this study highlights different mentorship approaches and their contributions to education in surgical residency programs. Moreover, this study summarizes the 10 stages of mentorship, offering a clearer understanding of the mentorship model in the context of graduate surgical education. Finally, the review provides insight into the common challenges and pitfalls among mentorship programs. The findings of this study aim to provide valuable guidance for developing effective mentorship programs in surgical residency programs, contributing to better support and outcomes for surgical trainees.

## Introduction and background

Mentorship can be traced back through history to Homer’s epic, The Odyssey [1-3]. Mentor is the human form of the goddess Athena, who takes care of Odysseus’ son Telemachus while Odysseus is at war in Troy. Today, mentorship has been described by surgical residents as an experience that extends beyond teacher-student interaction, with the mentor acting as a guardian and promotor of the young physician’s personal and professional development [3,4].

In the late 1800s, William Halsted created the first surgical residency at Johns Hopkins Hospital, envisioning a system in which surgeons hand-picked their apprentices for training [5,6]. Halsted’s original model has drastically changed over time, manifested today as “The Match,” which provides a more structured approach for continued education for new physicians, rather than simply pairing them with mentors [6-8].

Surgical education has a need for effective mentors at all levels of training [9,10]. Medical students working closely with residents develop relationships that influence their future career choice [11]; likewise, mentorship plays an influential role for residents when choosing a specialty [12,13]. An effective mentor can also lead to a more productive research career and greater job satisfaction in junior faculty [14,15]. The relationship that is formed between the mentor and mentee takes many forms. Often, mentees and mentors have different styles of learning and teaching. It is therefore important to discuss and classify the different approaches. The aim of this paper is to provide a comprehensive review of the literature and synthesize recommendations for identifying mentorship styles while avoiding common pitfalls throughout graduate surgical training.

## Review

Methods

Identifying and reviewing articles that met inclusion criteria involved two phases. During the first phase, we conducted a search of articles, yielding 68 articles using medical subject heading (MeSH) terms in PubMed, including residency, graduate medical education, mentorship, surgery, orthopedics, otolaryngology, plastic surgery, general surgery, vascular surgery, mentor, and mentee. Publications were included if they were published in English in a peer-reviewed publication and described mentorship in graduate surgical education.

Studies were excluded if they did not describe mentorship in surgical training, were limited to an abstract that lacked the detail necessary to evaluate surgical mentorship, or described a mentorship model that was specific to alternative fields of healthcare such as, but not limited to, psychology, nursing, and advanced care practitioners.

**Figure 1:**
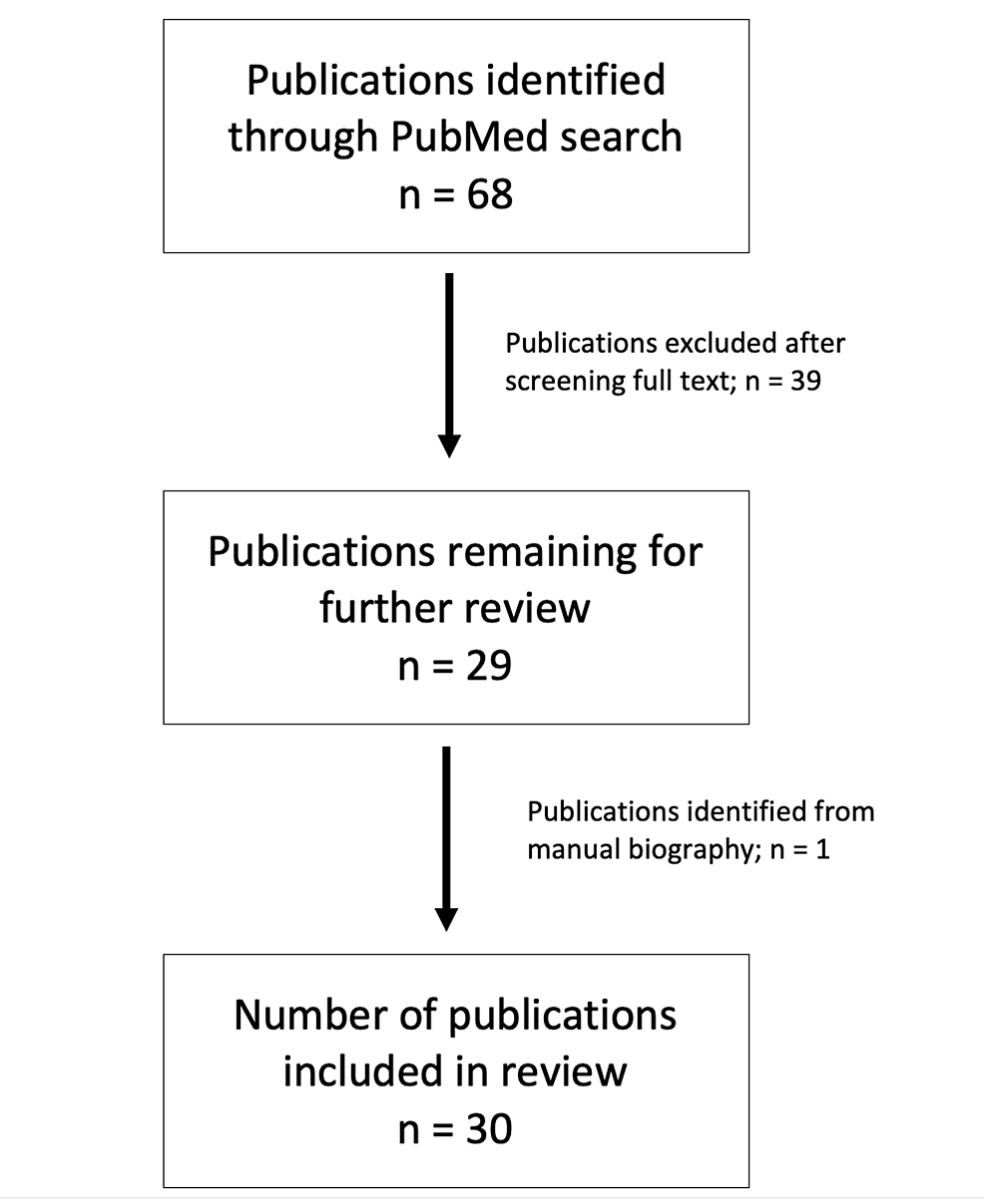
Article selection

A preliminary review of all articles for inclusion criteria was performed, with each author independently reviewing the full text of included publications to extract data regarding mentorship styles and common shortcomings of surgical mentorship (Figure 1). Based on the publications reviewed, a framework was developed for surgical educators to assess styles of mentorship to provide a road map for the development of future surgeons. Following an article review, the authors reviewed the data for comprehensiveness and consistency. A consensus was reached by utilization of the International Narrative Systematic Assessment Tool of the discussion portion of the manuscript [16]. The framework delineates various mentorship styles, including the following: near-peer, peer-to-peer, reverse, one-on-one, group, self-directed, alumni, and speed.

The initial search yielded 68 articles, of which 29 (43%) met the inclusion criteria. A manual search for reference sections identified one book chapter. From those publications, eight styles of mentorship were identified and described. In addition, 10 stages of mentorship progression were reported and summarized, to better elucidate mentorship steps within graduate surgical education. Finally, common pitfalls encountered during mentorship programs were explored in an effort to synthesize and present possible solutions to these problems. Figure 1 highlights the steps involved in our two-phase article selection method. 

Mentorship styles

Near-Peer

Definition: The mentor is a single step ahead of the mentee, such as a medical student receiving guidance from a first-year resident or intern.

Pros: The near-peer model may be best utilized during the transition to clinical years in medical training, which are traditionally perceived as the most stressful times in medical education, and is quite effective as the gap in the level of knowledge and training tends to be smaller [17].

Cons: The near-peer model is a less formal style of mentorship, so learning objectives for the mentee are crucial to achieving an effective education [17]. Among residents, negatives include extending work hours and an antagonistic work environment [5].

Best application setting: From the first few years of medical school continuing to early residency [17]. It can also be applied in surgical skill training, as younger surgeons still rely on a step-by-step method and not muscle memory [18].

Peer-to-Peer

Definition: The mentor and mentee are at approximately the same level of training or education.

Pros: More familiarity with the mentor, easy to establish a mentor-mentee relationship. Just as helpful as faculty or professor feedback [19].

Cons: Mentors lack the wide experience of knowledge typically employed by a more senior mentor. 

Best application setting: Mills et al. showed that third- and fourth-year dental students were able to motivate each other during various steps of patient treatment [19].

Reverse

Definition: A younger trainee who has specialized or unique knowledge and skill sets, assisting a more experienced individual, usually a more advanced resident or attending physician [20].

Pros: Older physicians can learn new skills essential to the continuation and refinement of their careers.

Cons: Reverse mentorship is rarely used in surgical specialties, or healthcare, in general, as senior-level physicians are so revered [20].

Best application setting: Reverse mentoring could be a useful tool as medicine becomes increasingly electronically oriented.

One-on-One

Definition: The most traditional form: one mentor, one mentee.

Pros: The benefits of this style cannot be over-emphasized; one-on-one teaching was found to be the preferred method of mentorship for medical students [21].

Cons: Not always feasible if a program does not have the resources to employ enough faculty.

Best application setting: Applicable in a variety of educational settings; even weekly one-on-one sessions provide adequate support to medical students [22].

Group

Definition: Exceeds the 1:1 ratio, one mentor to multiple mentees.

Pros: Utilizes a fewer number of senior-level mentors, which maximizes resources, educating the same number of students.

Cons: Less favored by medical students than a one-on-one mentorship style.

Best application setting: Students tend to require more than what surgeon mentors can provide, so group mentorship has become a necessity at smaller, less well-equipped institutions [21].

Self-Directed Mentorship (SDM)

Definition: The mentee does most of the work as the mentor provides general guidance and advice throughout the training and educational process.

Pros: SDM employs fewer instructional resources and a more efficient and effective approach to resident training [23]. 

Cons: It requires a highly motivated learner.

Best application setting: SDM is best used in conjunction with a simulation center, which historically is often under-utilized [23].

Alumni

Definition: Alumni from a given institution mentor recent graduates.

Pros: Supports graduates as they enter the professional world. Many schools have an extensive alumni network, which lends itself to this style of mentorship [24]. 

Cons: Not commonly used, possibly because of the concomitant presence of resident training for the mentees.

Best application setting: Alumni mentorship may be suited more toward professional students that do not go through post-graduate training [24].

Speed

Definition: Mentorships that are either a single day or long term, with short sessions lasting only a few minutes.

Pros: This form of mentorship aids the resident and mentor as they are frequently overwhelmed with time commitments.

Cons: Some learners may require more in-depth mentoring sessions to achieve their full potential.

Best application setting: Early clinical training; rapid teaching sessions between medical students and residents are considered beneficial to both parties [22].

Mentorship stages and effective traits

Mendler [25] outlined 10 different stages of relationship evolution in mentorship that were further expanded upon by Pellegrini (Table 1) [3].

**Table 1:** The 10 stages of mentorship

The 10 Stages of Mentorship	
Attraction	Pairing based on similarities or sought-after qualities in the mentor by the mentee
Cliché exchange	The initial meeting
Recounting	The sharing of experiences by the mentor
Personal disclosure	Each party discussing what they expect to gain from the relationship
Bonding	The two parties attempting to form a deeper connection
Fear of infringement	This signals a change in the relationship dynamic where the mentor senses that their protégé is transitioning into a colleague. A mentor with a secure ego is vital for advancing beyond this stage
Revisiting the framework	The mentor accepts the changing relationship dynamic and acknowledges it to the protégé
Peak mentoring	This stage depicts that a successful mentor-mentee relationship has taken place and has flourished. It is the beginning of the end of the relationship
Reciprocity	Clear mutual benefit to both mentor and mentee, both personally and professionally
Closure	The end of the relationship with satisfaction of both parties

The first five stages represent the initial relationship-building process between the mentor and mentee, including preliminary introductory meetings, recognizing personality similarities, and planning goals for the partnership. These stages often occur harmoniously, but by the fifth stage, the relationship can crumble due to ineffective mentoring [3]. Stages 6 and 7 represent how this relationship must be dynamic to succeed. The learner will progress, and the mentor must keep pace with this, or the mentorship will fail. Stages 8-10 represent the conclusion of the mentorship, which ideally results in a smooth transition for the learner to the next stage of their training and education.

Rowley suggests that there are six substantial traits that make a good mentor [26]. These include the following:

1) Commitment to the role of mentoring - Mentors should be engaged in the improvement of their mentees without personal gains or profits.

2) A sense of empathy - Mentors must appropriately assess the mentee’s level of experience while understanding the difficult process of medical education.

3) Skills in providing instructional support - Mentors should be refined teachers in their field while imparting their skills and wisdom to their learners.

4) Versatility in different interpersonal contexts - Mentors should be adaptable to a wide variety of learners and their individual needs. For example, resident education is different than medical student education, and a good mentor should understand this difference.

5) A modeled life of continuous learning - Mentors are able to instill strong core values of hard work and advancement of the mentorship cycle to future generations of learners.

6) The ability to communicate hope and optimism to their learner - Good mentors are not negative and do not unnecessarily berate their students.

Mentorship pitfalls and areas for improvement

A robust mentor-mentee relationship can be challenging to achieve. In a study by Flint et al., less than half of all the surveyed surgical residents were satisfied with their mentoring relationships [7]. One possible solution to increase satisfaction in the relationship is implementing a personality survey [27], which can give the mentor the ability to predict potential problems based on specific traits of their learner.

The lack of quality mentorship impacts surgical training at various levels of education and throughout multiple specialties. A recent survey of orthopedic surgery residents noted that, while 95% see value in a formal mentorship program, only 26% of respondents have a formal mentor [7]. This highlights the large discrepancy that is often seen in residency programs between the supply and demand of high-quality mentorship.

It is notable to mention that some authors believe that choosing a mentee is more of an art form, and not a decision that is necessarily based on resident test scores or clinical abilities [28]. Unfortunately, many surgeons do not always have personalities that are conducive to being successful mentors [7]. Becoming an excellent educator and mentor is a skill, and like any worthwhile endeavor, it requires patience, hard work, and practice. Surgical residents fortunate enough to have a mentor that possesses these traits will likely have a more satisfying and fulfilling relationship with their mentor. Not every educator placed in the position of mentor will have these qualities, and this can unfortunately result in a counterproductive mentorship. Pellegrini describes four different types of mentors for students to recognize and avoid (Table 2) [3].

**Table 2:** Pellegrini's four bad mentors

Pellegrini’s Four Bad Mentors	
The Uncommitted Phony	They are not involved in teaching and are easily distracted from their duties; look out for insincerity and dishonesty.
The Perfectionist-Turned-Tyrant	This individual leads by example and will initially seem like a great fit; do not be fooled. They will always expect perfection, and refuse to reinforce teaching with compliments, continuously raising expectations to near impossible standards. This relationship will quickly sour.
The Insecure Egocentric	This mentor-mentee relationship serves as an ego-builder for the mentor. Their insecurity will never allow for the relationship to flourish beyond the initial stages of mentorship.
The Begrudger	This mentor will be very protective of their own work and is extremely proud and self-righteous in their own path to success; they will only help the mentee by providing fragmented information in a cryptic and unhelpful manner.

Impediments to a successful mentorship program can also come from the core values of the mentor’s own institution or place of employment. Wilson discusses these obstructions [8]:

1) A culture that does not favor seeking help - Mentorship programs should strive to surround themselves with faculty and students who are willing to ask for help when needed.

2) Time constraints - Institutions should make it a priority to set aside time for the mentors to meet with their learners.

3) Providing resources to aid mentorship - It is difficult for one single individual to possess all of the tools necessary to be an effective mentor to every student at every level of their professional development. Institutional support ensures they have resources available to them to increase their repertoire of mentorship skills.

4) Lack of mentoring skills - Good mentors need to have enough foresight to ask thoughtful questions about their own abilities and the insight to not impose their own beliefs onto their mentees.

5) Lack of institutional support - Successful mentorship programs have visible support of their agenda by faculty in positions of power; discernable support should be evident at every level to convey the message of the importance of the mentorship program.

To be truly successful, mentoring needs to be viewed as a professional activity and should be formalized and treated like any other activity in medicine. Institutions involved in surgical training should set forth a formal mentoring program and ensure that their educators have the tools and the time needed to be excellent mentors, as well as ensure that their students understand what a good mentor is. Irrespective of the type of surgical training, residents will still seek to mimic good clinicians. Cultivation of future successful surgeons is imperative to the advancement of our surgical specialties and a good mentor realizes the credit for their efforts lies in the success of their student [29,30]. There is no amount of innovative educational replacements that can make up for the absence of a truly great mentor [5].

## Conclusions

Effective mentorship is a surprisingly difficult relationship, not only to form but to maintain throughout the length of a surgical residency. Medical education is a challenging journey, and this path is only more difficult without compassionate and caring leaders to help guide the way. There are numerous different styles of mentorship, some more appropriate than others in surgical education. Whatever the style, effective mentoring should be a dynamic relationship that evolves as the learner progresses in their own education. Both parties should have compatible personalities, and the mentor should possess traits that enable them to provide a healthy learning environment. The importance of quality surgical mentorship cannot be overstated, and surgical residency programs should make a definitive and continuous effort to provide this essential service for their residents.
